# HKR3 regulates cell cycle through the inhibition of hTERT in hepatocellular carcinoma cell lines

**DOI:** 10.7150/jca.39380

**Published:** 2020-02-10

**Authors:** Sung Hoon Choi, Kyung Joo Cho, Sung Ho Yun, Bora Jin, Ha Young Lee, Simon W Ro, Do Young Kim, Sang Hoon Ahn, Kwang-hyub Han, Jun Yong Park

**Affiliations:** 1 Yonsei Liver Center, Yonsei University College of Medicine, Seoul, Republic of Korea; 2 BK21 plus project for medical science, Yonsei University College of Medicine, Seoul, Republic of Korea; 3 Division of Bioconvergence Analysis, Drug & Disease Target Team, Korea Basic Science Institute (KBSI), Cheongju, Republic of Korea; 4 Bio-Analysis Science, University of Science & Technology (UST), 217 Gajeong-ro, Yuseong-gu, Daejeon, Republic of Korea; 5 Department of Internal Medicine, Institute of Gastroenterology, Yonsei University College of Medicine, Seoul, Republic of Korea

**Keywords:** Hepatocellular carcinoma, Cell cycle, CDKN2A, hTERT, HKR3

## Abstract

Hepatocellular carcinoma is a malignant disease with improved hepatic regeneration and survival, and is activated by human telomere transferase (hTERT). hTERT is expressed during early fetal development and switched off in most adult tissues, but it becomes reactivated in HCC. The exact mechanism regulating these expression changes remains unknown during HCC progress. We evaluated the relationship between hTERT expression and human kruppel-related 3 (HKR3) and cell cycle-related factors in HCC cell lines. Following transfection for hTERT knockdown and HKR3 overexpression, proteomic and transcriptomic analyses related to hTERT were performed using liquid chromatography/mass spectrometry (LC/MS) and RNA sequencing (RNAseq) in HCC cell lines. The expression levels of hTERT, HKR3, and cell cycle-related factors were measured using western blotting, and tumor growth were evaluated via cell proliferation and cell cycle assays. Transcriptomic and proteomic analyses showed that HKR3, hTERT and cyclin-dependent kinase inhibitor 2A (CDKN2A) were correlated. Up-regulation of HKR3 expression decreased hTERT and cyclin activation and suppressed the G1/S phase of the cell cycle through CDKN2A activation. Our results suggest that HKR3 induced regulation of cell cycle through hTERT inhibition and CDKN2A activation. Our results will facilitate further exploration of the pathways regulating human telomerase activity in HCC cell lines.

## Background

Hepatocellular carcinoma (HCC) is the sixth most common cancer worldwide and forms malignant tumors with poor prognosis 1,2. Its progression is marked by the sequential accumulation of genetic and epigenetic abnormalities that give rise to a set of common characteristics including the ability to promote cell proliferation and escape apoptosis 2. Loss of sensitivity to apoptosis and up-regulation of the cell cycle contribute to tumor progression and metastasis and determine how cancer cells respond to treatment 3. Therefore, deciphering how the cell cycle and apoptosis are regulated in HCC cells is important to understanding how these tumors develop and improving their clinical management 4.

The regulation of cell proliferation is a complex process involving numerous transcription factors and regulatory proteins 5. HCC proliferation is controlled by the dynamic regulation of the expression of oncogenes and tumor suppressor genes in response to various signals 5,6. Telomerase plays a key role in conferring immortality to cancer cells through the regulation of telomere length 7; it synthesizes telomeric repeats at the ends of chromosomes and replaces end sequences that are progressively lost during each cell cycle, allowing cells to escape mortality and continue to proliferate 7,8. Reverse transcriptase telomerase is composed of two core components, a ubiquitously expressed RNA component and a catalytic subunit, human telomerase reverse transcriptase (hTERT) 8,9, whose expression is limited to the formation of a catalytically active enzyme and the regulation of telomerase activity 10,11. Increased telomerase activity (TA) is observed in 90% of human cancer cells 8,9; however, telomerase activity is low or undetectable in most normal human somatic cells 9. Therefore, inhibition of hTERT could be an effective anti-tumor strategy in HCC treatment.

Previous studies on several cancer cell lines have indicated that inhibiting telomerase, particularly hTERT, using antisense RNAi is highly promising as a cancer therapy 12. Plasmid-encoding hTERT-specific shRNAs have been transfected into human hepatocellular carcinoma cell lines and have been found to stably suppress hTERT expression 12,13, leading to the inhibition of cell proliferation and attenuation of tumorigenic potency 14. The transfection of encoding hTERT-specific siRNAs into human breast cancer cell lines has been shown to inhibit cell proliferation and induce cell apoptosis 15. Recently, several studies have also proved that the knockdown of hTERT effectively inhibits the expression of telomerase activity, cell proliferation, and apoptosis in variable tumor cells in vitro 12,14-16.

As the role of hTERT in HCC increases, studies on factors that correlate with hTERT are actively proceeding. Although it was confirmed that steroidogenic acute regulatory protein **(**StAR) was the factor that increased the expression of hTERT, the negative activity of hTERT is regulated by HKR3 (ZBTB48), but it is not clear 17. HKR3 is a relatively uncharacterized POK family protein with a POZ-domain and 11 zinc finger domains 18,19. HKR3 is ubiquitously expressed in human tissues 20. The HKR3 gene is mapped to human chromosome 1p36, which is commonly rearranged (leiomyoma and leukemias) or deleted (neuroblastoma, melanoma, Merkel cell carcinomas, pheochromocytoma, and breast and colon carcinomas) in various cancers 19,21. A correlation between deletions or rearrangements of HKR3 and human cancer suggests a role for HKR3 as a potential tumor suppressor 19,21.

Since expression of hTERT is highly maintained in HCC, the expression of co-related factors involved in survival or growth by hTERT remains high 22. As well, although the cell survival in phenomena such as regulation of apoptosis by hTERT is known 11, those involving hTERT and HKR3 remain poorly understood in HCC. Therefore, we examined the effects of hTERT knockdown and HKR3 overexpression on cell proliferation, cell apoptosis, and the cell cycle, and evaluated the relationships between hTERT expression and both HKR3 and CDKN2A in HCC cell lines.

## Materials and Methods

### Patient samples

Tumor tissues and matched adjacent non-tumor liver tissues were obtained with informed consent from HCC patients who underwent curative resection between 2012 and 2017 at Severence Hospital (Yonsei University College of Medicine, Seoul, Republic of Korea). A total of 42 pairs of primary HCC tumor tissues and matched normal tissues were used for real time quantitative PCR, immunohistochemistry. The study was conducted in accordance with the ethical guidelines of the Declaration of Helsinki and approved by the Ethics Committee of Severence Hospital.

### Cell culture

Huh-7(KCLB60104, Korean Cell line Bank), Hep3B (KCLB88064, Korean Cell line Bank), and HepG2(KCLB88065, Korean Cell line Bank) cells were cultured at 37°C with 5% CO_2_ in Dulbecco's Modified Eagle Medium (DMEM; Gibco, Grand Island, NY) supplemented with 10% fetal bovine serum (FBS; Gibco, CA, USA), 4.5 g/L glucose, L-glutamine, and 1% penicillin/streptomycin.

### small interfering RNA (siRNA), Plasmid construction and transfection

siRNA was synthesized using the following sequences: siTERT #1: (Forward) 5'-GUC UUC CUA CGC UUC AUG Utt-3', (Reverse) 5'-ACA UGA AGC GUA GGA AGA Ctt-3' (Santa cruz, CA, USA). This vector is pcDNA3.1, which is generally used to induce the expression of a specific sequence. We constructed a plasmid for overexpressing full length HKR3 (NM_001278647.1). Cells were transfected with respective siRNAs using Fugene HD transfection agent (Promega, Madison, WI, USA) according to the manufacturer's instructions. After transfection, the cells were incubated at normal conditions (21% O_2_) for 6 h, then replaced with fresh culture medium.

### Cell Growth Assay

HCC cell growth rates were measured using the WST-1 (2-(2-methoxy-4-nitrophenyl)-3-(4-nitrophenyl)-5-(2,4-disulfophenyl)-2H-tetrazolium (WST-1, Sigma-Aldrich, CA, USA) method. First, the cells were seeded in a 96-well plate at 5 x 10^3^ cells/well, incubated at 37°C for 24 hours, transferred to serum-free medium, and transfected with siRNA as described above. Then, after cultivation under 21% O_2_ for 24 hours, the cells were transferred to a culture medium containing 10% FBS. Finally, WST-1 was added to the wells, and the plates were incubated at 37°C for 3~4 hours for efficient cell dyeing, and analyzed for its absorbance at 460 nm using a spectrophotometer (Molecular Devices, USA).

### Cell cycle and death assay - FACs

Cells were stained with FITC-labeled annexin V and propidium iodide, and tumor cell death was assessed by terminal deoxynucleotidyl transferase dUTP nick end labeling (TUNEL; Millipore, Billerica, MA, USA) assay and flow cytometry (BD- Flow JO). Cell cycle samples also were stained propidium iodide (PI) staining) after EtOH fixation at 4^o^C, O/N.

### mRNA isolation and real-time RT-PCR

For real-time reverse transcription PCR (RT-PCR) studies, cells were seeded in 6-well plates until complete adhesion (85% to 90% confluence) and then incubated overnight in serum conditions. mRNA levels of human TERT, HKR3 and cell cycle factors were determined using SYBR Green-based semi-quantitative PCR. RNA was extracted using a QIAGEN Rneasy mini kit (QIAGEN, Hilden, Germany) according to the manufacturer's instructions and reverse-transcribed (RT) using a Clontech RT Kit (Clontech, Terra-bella, CA, USA). After the RT reaction, qPCR analysis was performed using an applied biosystem syber green master mix and specific PCR primers (Table S1). Amplification efficiencies were calculated for all primers utilizing serial dilutions of the pooled cDNA samples. The data were calculated, using the comparative (ΔΔCt) method, as the ratio of each gene to the expression of beta actin, the housekeeping gene. Data are shown as mean ± s.e.m. (error bars) of at least three independent experiments and represented as fold-change vs. controls. Melting curves were generated to ensure a single gene-specific peak, and no-template controls were included for each run and each set of primers to control for unspecific amplifications.

### Immuno-blot analysis

The effects of inhibition of hTERT expression on the expression of proteins related to cell cycle and apoptosis were assessed using immuno-blot. Total proteins were collected from siTERT and HKR3 transfected HCC cells after they were incubated under culture conditions for 24 hours and lysed. The proteins were separated according to their molecular weight via sodium dodecyl sulfate-polyacrylamide gel electrophoresis, transferred to a polyvinylidene fluoride membrane (GE Healthcare, Amersham, Buckinghamshire, UK), and probed with mouse monoclonal antibodies specific for the proteins of interest. The blots were developed using the ECL technique (PerkinElmer, Boston, MA, USA) according to the manufacturer's instructions, and the level of expression of each protein was quantified and compared (hTERT, HKR3, CDKN2A and CDKN2B: Invitrogen, Cambridge, MA, USA; Cyclin B1, Cyclin D1 and Cyclin E1 : Cell signaling, Danvers, MA, USA; CDK2 and CDK4: Santa cruz, CA, USA). For the detection of cellular proteins as hTERT, an enzyme-linked immunosorbent assay (ELISA; R&D Systems, CA, USA) was performed according to the manufacturer's instructions. IHC also used same antibodies. The obtained sections were subjected to dewaxing in xylene and dehydration in several graded ethanols, then heated to 100 °C in 0.1 M citrate buffer (pH 6.0) for 30 min to retrieve the antigens. 3% H_2_O^2^ was used to block the activity of endogenous peroxidase for 10 min, then incubated with the indicated primary antibody (anti-TERT and anti-HKR3) overnight at 4 °C. After washing with PBST (with 0.5% Tween-20), xenograft or patients tissues were incubated with secondary antibodies, comprising horseradish peroxidase-conjugated anti-rabbit or anti-mouse IgG (Envision kit, Dako, Denmark). Finally, the hematoxylin-stained sections were observed under an Axio Observer A1 microscope (Carl Zeiss, Jena, Germany).

Transcriptomic and proteomic analyzes were confirmed by RNAseq and LC / MS. The above analysis was performed as described in Supporting Materials and Methods.

### Statistical analysis

Results were expressed as means ± standard error of the mean (SEM) or frequency (%). An independent *t*-test was performed to compare the difference of the means between control and experimental groups. All statistical analysis was done using SPSS version 12.0 (SPSS, Inc., Chicago, IL). A *p* value of less than 0.05 was considered statistically significant.

## Results

### 1. hTERT is highly expressed in HCC

**Figure 1:**
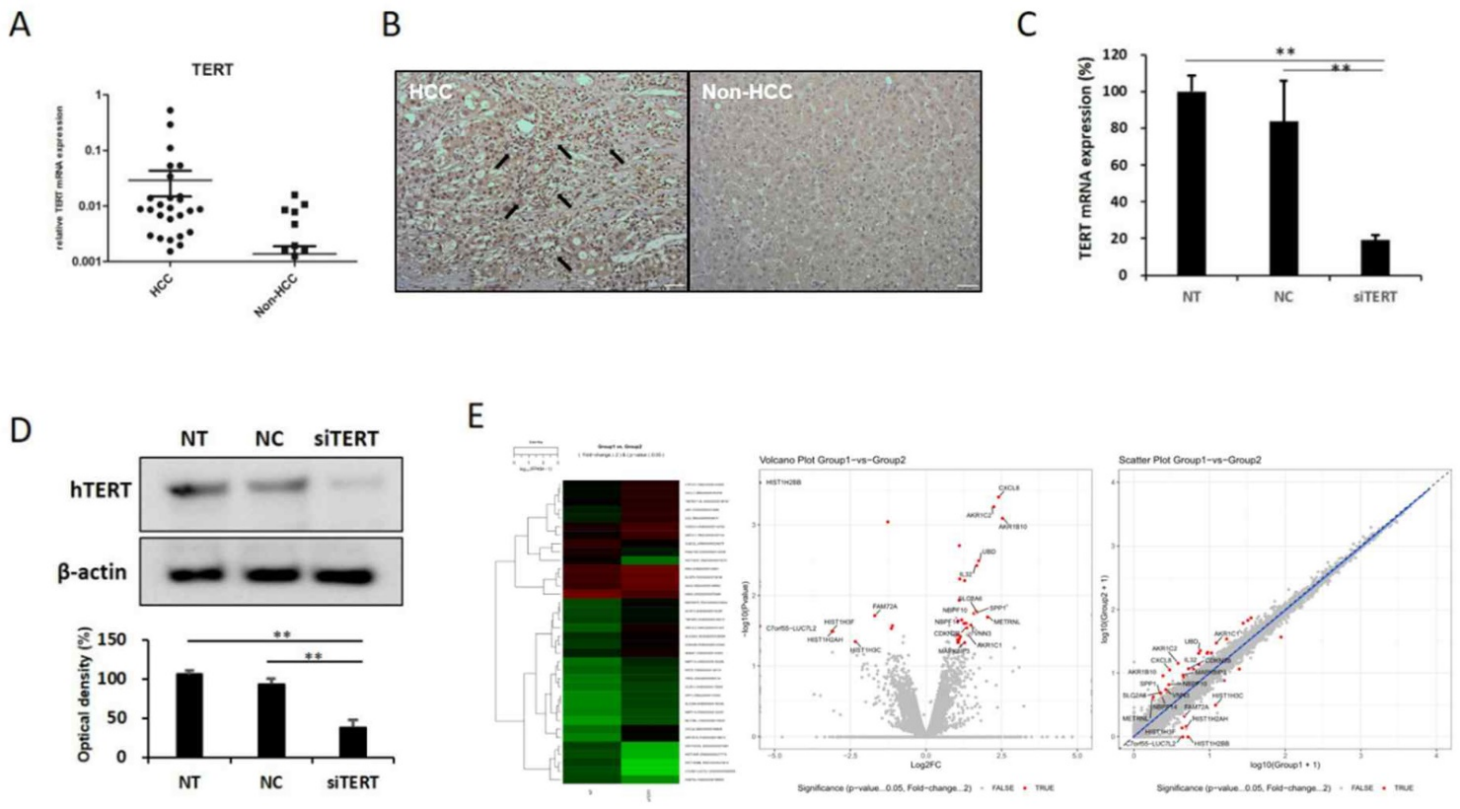
** hTERT expression and its correlation with HCC.** hTERT was overexpressed in HCC. (A) hTERT expression in tissues of 42 HCC patients was confirmed by real-time quantitative polymerase chain reaction (qPCR). Expression was higher in the HCC group than in the non-HCC group. (B) Immunohistochemistry results also showed that hTERT expression was higher in the HCC group than in the non-HCC group. (C, D) siTERT activity was confirmed by qPCR and Western blotting. hTERT expression was inhibited by ≥80% (NC vs *p < 0.05, **p < 0.001). All experiments were repeated three times. (E) Heatmap, volcano plot, and scatter plot of the 40 most differentially expressed genes between control and hTERT knockdown Hep3B cells determined by RNA sequencing (RNAseq) analyses (≥2 fold change, p ≤ 0.05).

In order to identify factors controlling hTERT, we first examined the expression pattern of hTERT in HCC tissues. To confirm increased hTERT expression in HCC, 42 patients with hepatocellular carcinoma underwent rt-qPCR. HCC tissues showed approximately 15 times more hTERT expression than normal tissues (Fig. 1A), and immunohistochemistry (IHC) results also indicated increased hTERT expression in HCC tissues (Fig. 1B). Based on these results, most of the patients with HCC showed high expression of hTERT.

### 2. Identification of hTERT related factors by transcriptomic analysis

**Figure 2:**
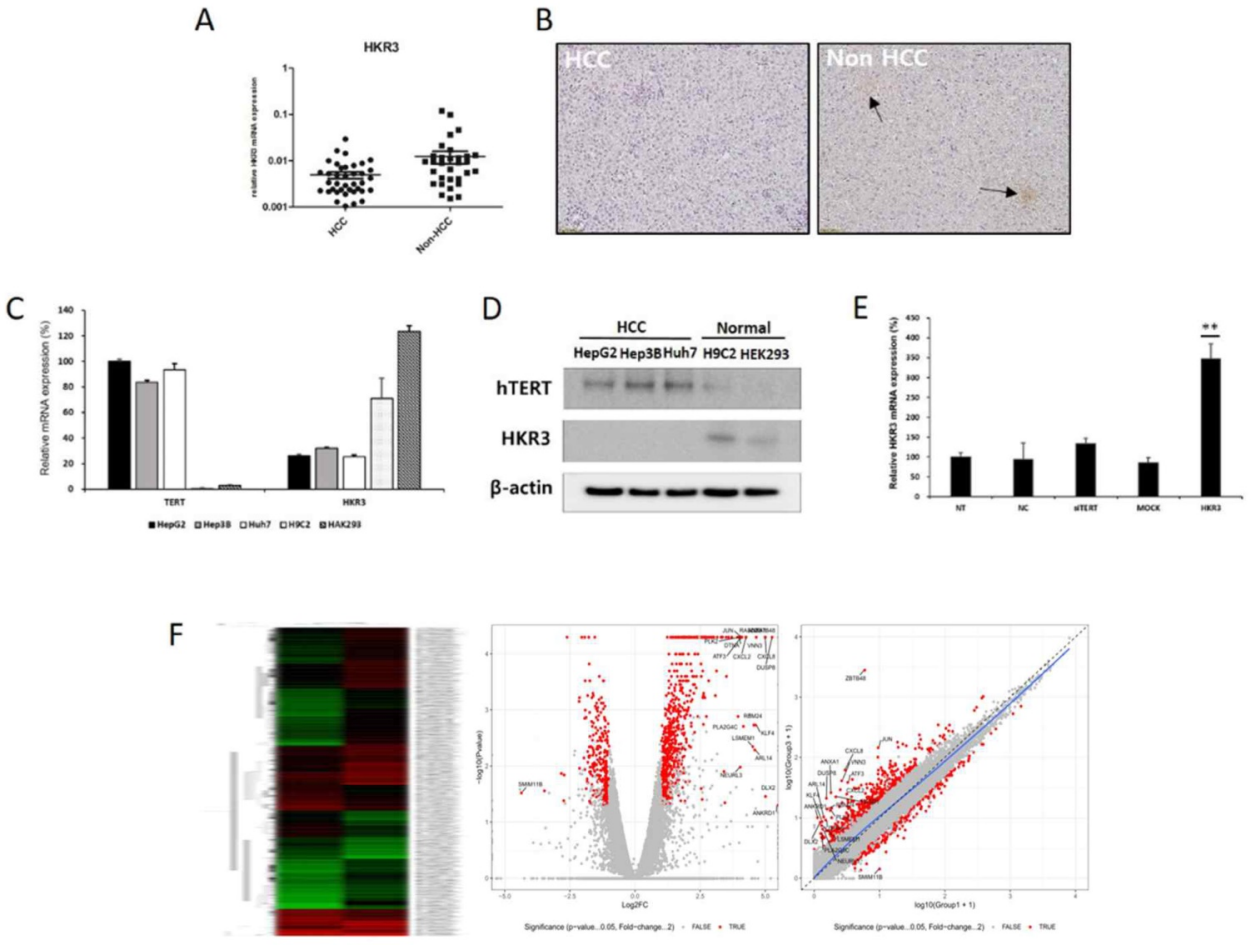
** HKR3 expression and hTERT regulation.** (A) HKR3 expression in tissues of 42 HCC patients was confirmed by real-time qPCR. hTERT expression was higher in the non-HCC group than in the HCC group. (B) Immunohistochemistry results also showed that HKR3 expression was higher in the non-HCC than in the HCC group. (C, D) hTERT and HKR3 expression was confirmed in various cell lines. The results of qPCR and Western blotting showed high hTERT expression in liver cancer cell lines HepG2, Hep3B, and Huh7, and high HKR3 expression in normal cell lines H9C2 and HEK293. All experiments were repeated three times. (E) siTERT activity and HKR3 overexpression were confirmed by rt-qPCR. HKR3 expression was increased by ≥ 4 times (NC vs or Empty vs *p < 0.05, **p < 0.001). All experiments were repeated three times. (F) Heatmap, volcano plot, and scatter plot of the 944 most differentially expressed genes between control and hTERT knockdown Hep3B cells determined by RNAseq (≥2 fold change, p ≤ 0.05).

In order to investigate the factors regulating the expression and activity of hTERT, and it was inhibited using small interfering hTERT (siTERT). (Figure 1C, D). After suppression of hTERT expression, changes in mRNA expression were analyzed via RNAseq. Although changes in genes related to apoptosis were the most frequent, we also observed changes in cell cycles and senescence-related genes (Figure 1E, Figure S1A). So, we also detected genes controlling hTERT expression and confirmed that HKR3 and hTERT were correlated. Consequently, rt-qPCR results showed that hTERT expression occurred more frequently in non-HCC than in HCC (Figure 2A). However, IHC results revealed almost no HKR3 expression within cancer tissues of HCC patients; instead, HKR3 was found mainly around the bad prognosis of liver tissue, not HCC (Figure 2B). When hTERT and HKR3 expression were compared in cell strains, hTERT was more evident in cell strains of HCC, whereas HKR3 appeared more frequently normal cell strains (Figure 2C, D). After confirmation of HKR3 overexpression and hTERT knockdown (Figure 2E), analysis of hTERT-derived genes in HKR3 overexpression based on RNAseq analysis yielded similar pattern of hTERT knockdown. The cell cycle and cell death-related changes are 26-29% of the total changes, and other changes such as cell communication, metabolic process, cellular components can be identified (Figure S1B). In other words, we observed gene expression related to apoptosis and changes in gene expression related to the cell cycle (Figure 2F), indicating that the genes involved with HKR3 and hTERT are closely correlated.

### 3. Verification of transcriptomic results for hTERT and HKR3 by proteomic analysis

**Figure 3:**
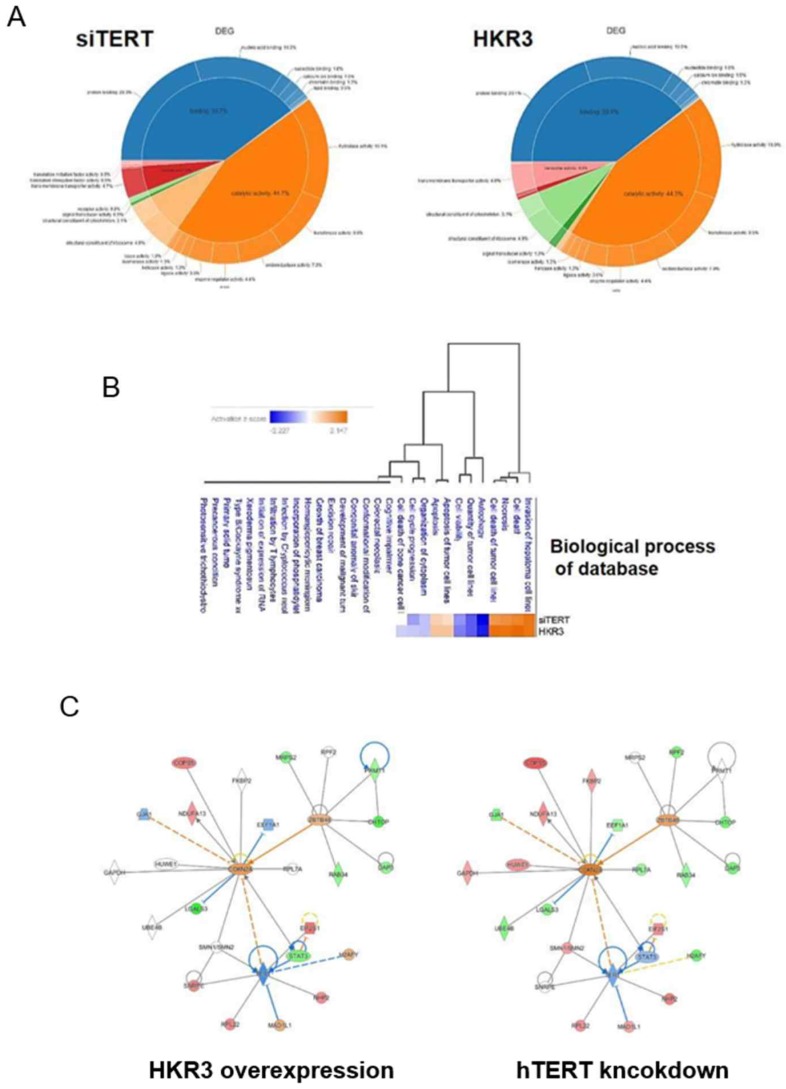
** Proteomic analyses of hTERT and HKR3.** We performed ingenuity pathway analyses (IPA) of 268 differentially expressed proteins identified from a comparison between siTERT and HKR3 overexpression groups using an edge rank algorithm. (A) Protein functions of siTERT and HKR3 were showed by Venn diagram (B) A network for biological process of connected pathways identified between siTERT and HKR3 by liquid chromatography/mass spectrometery (LC/MS). Red, upregulated; blue, downregulated. (C) Other interacting molecules (Red: up-regulation, Green: down-regulation, Orange: Predicted as up-regulation, Blue: Predicted as down-regulation)

To verify consistency between the results of dielectric and proteome analyses, we conducted LC/MS analyses. A total of 3,500 proteins were detected in HCC cell lines. Of these, 1,408 (40.2%) proteins were commonly expressed in each groups. By contrast, 625 and 567 were expressed uniquely in control groups (NT and NC groups) and experimental groups (HKR3 and siTERT groups), respectively (Figure S1C). In addition, the expression levels of 585 of the 2,127 proteins in HKR3 groups and 851 of the 2,486 proteins in siTERT groups were altered by at least 2.0-fold compared to NC groups, or uniquely identified. Proteins with an expression level of mol % > 0.001 were selected for DEG analysis and used for post analysis. Similar patterns of protein expression among the siTERT group and the HKR3 over-expression group were obtained (Figure 3A). Proteomic analysis shows that binding and catalytic activity are similar patterns. On the other hand, different patterns can be identified in structure, transport and signal transducer. Based on these results, we observed a change in protein expression that was analogous to that observed in our RNAseq dielectric analyses (Figure 3B). Apoptosis or survival related gene changes were most consistent in the same results of RNAseq and proteomic analysis. Subsequently, analysis of changes in cell structure, cell cycle and cell proliferation was highly consistent. Based on results of the cell cycle, cell proliferation and cell death, Ingenuity pathway analyses (IPA) of the protein network in the hTERT suppression/HKR3 overexpression group showed that hTERT is regulated by STAT3, is associated with cell differentiation factors, and predicts to modulate CDKN2A. HKR3 is associated with cell respiration, apoptosis factors, and also regulated CDKN2A (Figure 3C). Interestingly, both HKR3 and hTERT were related to the vitalization of CDKN2A in the cell cycle.

### 4. HKR3 inhibits hTERT expression and activation of CDKN2A

**Figure 4:**
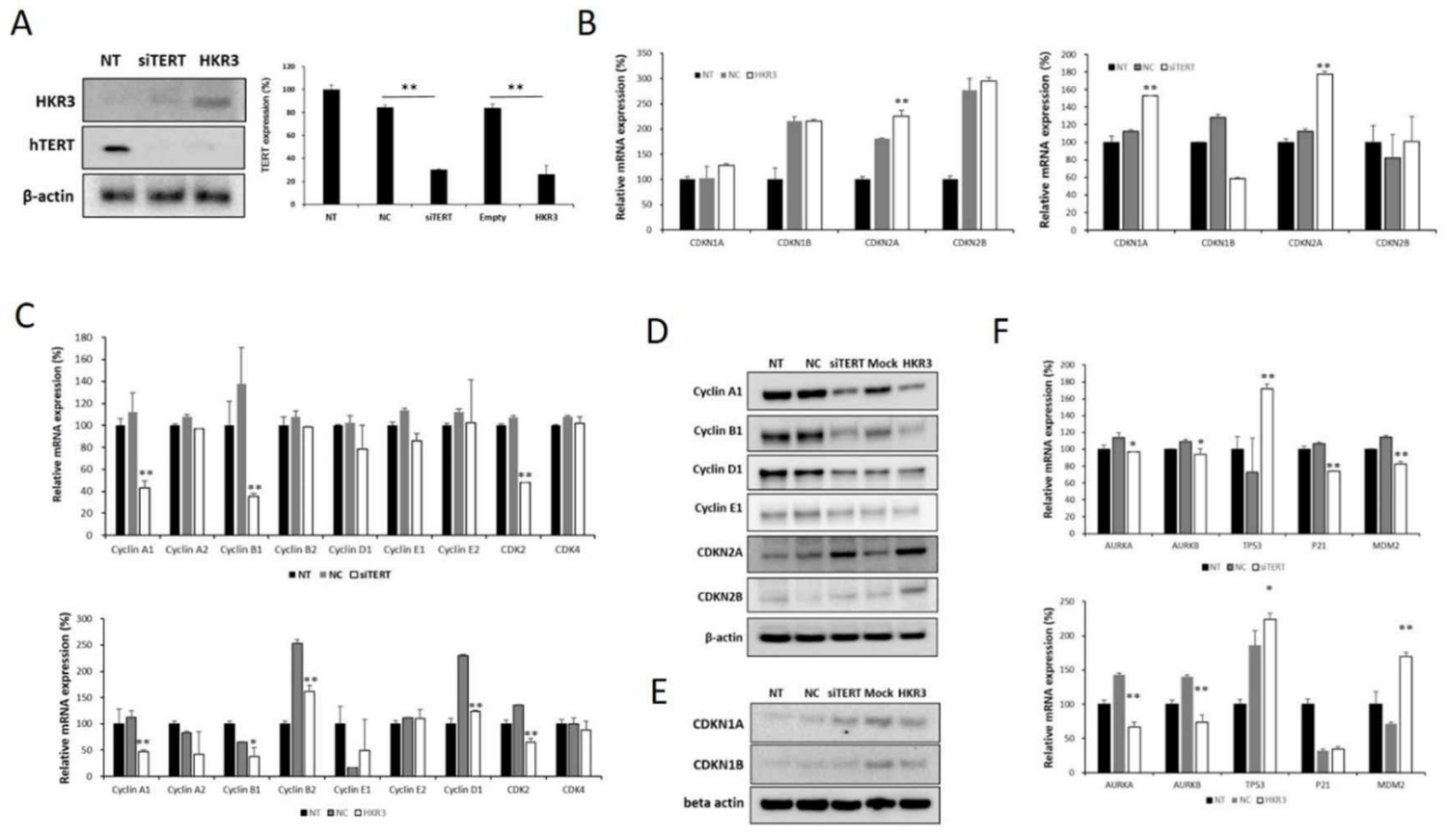
** Changes in cell cycle factors identified by hTERT knockdown and HKR3 overexpression.** We confirmed the findings of our transcriptomic and proteomic analyses by performing qPCR and Western blotting. (A) hTERT and HKR3 expression in the siTERT and HKR3 overexpression groups was confirmed by Western blotting; hTERT expression decreased in both groups. (B) The expression of cell cycle factors in the siTERT and HKR3 overexpression groups was confirmed by qPCR. CDKN1A and CDKN2A expression was higher in the hTERT suppression and HKR3 overexpression groups than in the control group (NC vs or Empty vs *p < 0.05, **p < 0.001). (C) In siTERT and HKR3 overexpression, expression of cyclins and cyclin dependent kianses (CDKs) was detected and confirmed by rt-qPCR (NC vs or Empty vs *p < 0.05, **p < 0.001). (D) Western blotting results indicated that CDKN2A expression was higher in the hTERT knockdown and HKR3 overexpression groups than in the control group. However, cyclin A1 and B1 were higher in the control group than in the hTERT knockdown and HKR3 overexpression groups. (E) CDKN1 slightly increased in siTERT and HKR3 compared to control. (F) In siTERT and HKR3 overexpression, expression of AURK, p53, p21 and MDM2 was detected and confirmed by rt-qPCR (NC vs or Empty vs *p < 0.05, **p < 0.001). All experiments were repeated three times.

Factors related to the cell cycle were verified in the hTERT knockdown and HKR3 expression groups based on results of the transcriptomic and proteomic analyses. hTERT expression decreased with increases in HKR3 expression in the siTERT and HKR3 overexpression groups (Figure 4A). We confirmed mRNA expression in factors related to the cell cycle through qPCR and observed that CDKN2A expression was increased (Figure 4B), whereas the expressions of cyclin A1, B1, and D1, which are controlled by CDKN2A, were reduced (Figure 4C). Both CDKN2A groups showed an upward trend in protein expression, whereas cyclin A, B, and D experienced decreased expression (Figure 4D). In addition, CDKN1A, which showed a slight increase in rt- qPCR results (Figure 4B), also slightly increased (Figure 4E). We also confirmed that p53, p21, and MDM2, which is the major tumor suppressor protein involved in the cell cycle, was correlated with hTERT or HKR3 expression. As a result, p53 was significant with siTERT or HKR3 group, while p21 and MDM2 were not significant. On the other hand, Aurora kinase A and B (AURKA and AURKB), the cell cycle accelerate protein, were significantly decreased in hTERT knockdown or HKR3 overexpression group (Figure 4F). Based on these results, hTERT is suppressed by HKR3, and CDKN2A is activated by the suppressed hTERT.

### 5. CDKN2A inhibits cell cycle arrest in HCC by inhibiting hTERT via HKR3

**Figure 5:**
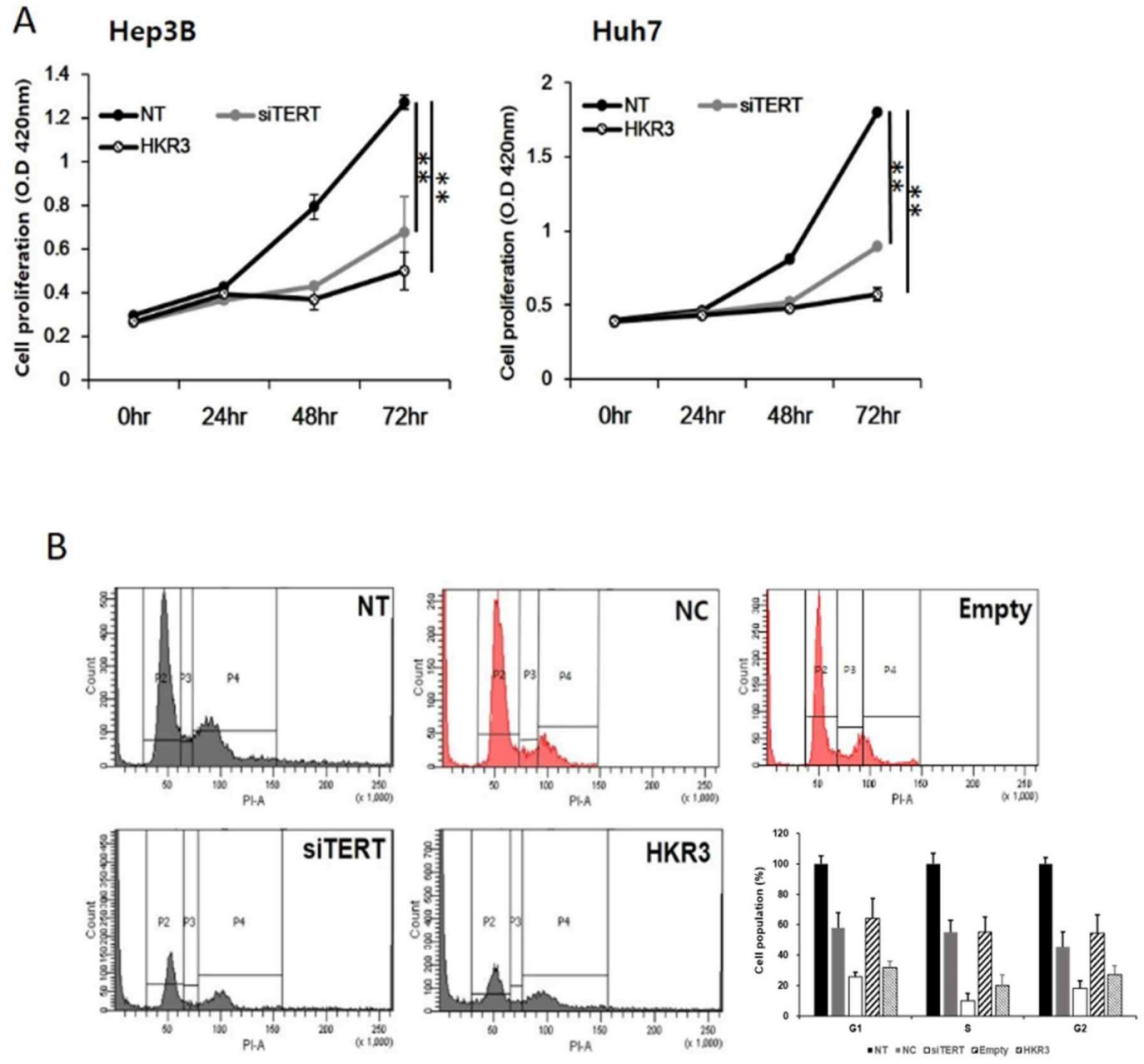
** Regulation of cell cycle and cell proliferation by hTERT knockdown and HKR3 overexpression.** (A) The inhibition of cell proliferation was greater in the siTERT and HKR3 overexpression groups than in the control group, in a time-dependent manner. The inhibitory effects of HKR3 overexpression were greater than those of the hTERT knockdown group (NC vs or Empty vs *p < 0.05, **p < 0.001). (B) FACs-PI staining was performed to confirm cell cycle arrest by siTERT and HKR3. Cell cycle arrest in the G1/S phase was observed to a greater extent in hTERT knockdown and HKR3 overexpression groups than in the control group. All experiments were repeated three times.

As the results of the protein and mRNA-level analyses, we performed cell proliferation and cell cycle assays and found that hTERT and HKR3 controlled the cell cycle. Cell proliferation was inhibited in both siTERT and HKR3 overexpression groups in Hep3B and Huh7, time-dependently (Figure 5A). Both groups showed inhibitory effects after 24 hours. In HCC cell lines, about 50% inhibition effect was confirmed in the siTERT treated group and the inhibitory effect of about 70% was confirmed in HKR over-expression group. Furthermore, both groups showed cell cycle arrest in the G1/S phase (Figure 5B). Based on these results, we conclude that the cell cycle was inhibited by CDKN2A when hTERT expression was suppressed or HKR3 expression increased.

## Discussion

HCC is a malignant disease belonging to a high risk group which is difficult to treat due to various mutations and drug metabolic functions of the liver. In particular, hepatocellular carcinoma is difficult to treat due to many obstacles characterized by heterozygous mutations. Especially *hTERT* promoter mutation, which is one of the mutations, has a great influence on the progress of HCC 23,24. Besides, Regenerative tissues such as the liver are similarly regulated in tumors using hTERT, STAT, MAPK signal, et al. 25. On the other hand, HKR3 inhibited the expression of hTERT and decreased telomere activity in early cancer 17. Inhibition of hTERT has been reported to inhibit cyclin activity 26. Therefore, the correlation between hTERT, HKR3 and cell cycle factors can be confirmed. Peculiarly, hTERT-mediated cell cycle regulation in HCC has a great effect on tumor viability. Therefore, since various hTERT signals are not well known, studies on HCC and hTERT are being performed continuously. Based on this fact, we detected the overexpression of hTERT in HCC cells. The results revealed an arrest of cell cycle and an increase of apoptosis induced by the knockdown of hTERT and HKR3 overexpression in HCC cells, indicating that the down-regulation of hTERT had the potential anticancer effects in HCC. Therefore, studies on the regulation of hTERT have been conducted in the development of anticancer drugs and therapies in HCC.

Several studies have explored the correlation between hTERT and tumors such as leukemia, cervical cancer, breast cancer, and HCC 8,14,15,22,27. Aging of natural cells through reduction of telomere causes apoptosis, or apoptosis also occurs through hTERT inhibition. Thus, over-expression of hTERT leads to the synthesis of telomeres to prevent apoptosis in most solid tumors 11. However, whether hTERT leads the cell cycle by inhibiting factors other than telomeres, including CDKN2A, has remained unknown. The current study demonstrated that hTERT expression is not limited to anti-apoptosis and tumor survival but also affects the tumor cell cycle 11,14. We also confirmed that inhibition of hTERT induces apoptosis (Figure S2). So, we investigated the mechanism controlling the cell cycle using the link connecting HKR3, which regulates hTERT, to CDKN2A, which is regulated by hTERT through transcriptomic and proteomic analysis.

Changes in gene expression following the suppression of hTERT expression were observed in RNAseq analyses. Among these changes, the major one is apoptosis, with genes related to cell cycle progression and cytoplasm organization showing the most changes. Differentially expressed gene analyses were conducted to explore telomere-related genes including StAR, hTERT-related gene that up-regulates HDAC and hTERT, and HKR3, which inhibits hTERT expression. Changes in gene expression are induced by suppressing hTERT expression and overexpressing HKR3; we observed a similar pattern of gene changes in this study. Most changes occurred in genes related to apoptosis, followed by those involved in the cell cycle.

For background studies on the variable disease, many researchers carry out RNAseq or next-generation sequencing (NGS), a transcriptomic analysis 28-30. Other researchers perform proteomic analysis by considering protein modification more important than gene expression 31,32. Most of the expression of the genome is linked to the activity of the protein, but it may be removed before the protein is activated, or it may function differently from its original role 33. On the other hand, very few genomic expressions have potent protein activity 34. It may be over-expressed in certain conditions and may be involved in various biological processes due to intracellular accumulation such as HIF by hypoxia 35. The results were similar to those of transcriptomic analyses, with similar patterns observed in protein changes associated with apoptosis, the cell cycle, and cell structure.

Similar changes in protein expression were observed in the hTERT inhibition and HKR3 overexpression groups. However, the regulation of HKR3 expression involved more gene and protein changes in more areas compared to those for hTERT, which is involved in telomere synthesis in the nucleus. This differential expression can be explained by the fact that HKR3 is a telomeric zinc finger-associated protein that controls telomere elongation and acts as a transcription activator 19, regulating the expression of various genes. Previous studies have reported that HKR3 inhibits ARF expression and cell proliferation in the ARF region 36,37, indicating that among various tumor-suppressing functions, HKR3 also acts as a regulator of the cell cycle, which is controlled by ARF and cell proliferation 19,36,38,39.

**Figure 6:**
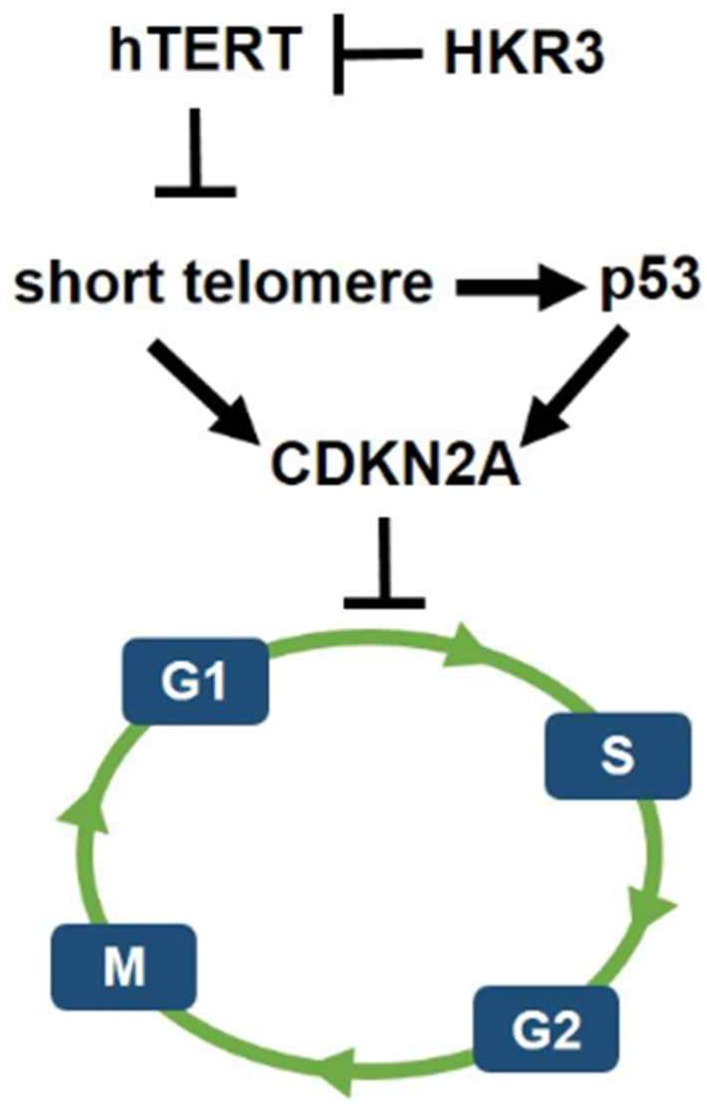
** Schematic of hypothetical HKR3 signaling cascade.** Mechanistically, short telomeres activate p53 that induces cell cycle arrest. Blunting telomere shortening via overexpression of hTERT. And, HKR3 inhibits the expression of hTERT and binds telomere to inhibit the activity of hTERT.

However, less research has been conducted on HKR3 than on hTERT. Thus, this result confirms the correlation between HKR3 and hTERT, thereby establishing a linkage that regulates the cell cycle. Namely, the cell cycle and apoptosis of HCC were regulated by hTERT. In addition, it was confirmed that hTERT expression was inhibited by HKR3. Moreover, p53 is activated by the short telomere, which leads to cell cycle arrest 40. Therefore, HKR3 regulates cell cycle and apoptosis of HCC through regulation of TERT (Fig. 6). Based on these results, further studies of the mechanism by which HKR3 expression is suppressed in HCC and the roles of HKR3 in transcription activation and HCC progression are required. These studies will be used as background data for clinical application of recombinant proteins such as HKR3 in HCC. We also anticipate that our results will be used to promote treatment that can prevent the HCC progress through the inhibition of hTERT using HKR3.

## Supplementary Material

Supplementary figures and table.

## Abbreviations

hTERT: human telomere reverse transcriptase
TR: telomerase activity
StAR: Steroidogenic acute regulatory protein
HKR3: Human Krüppel-related 3
RNAseq: RNA sequencing analysis
LC/MS: Liquid Chromatography / Mass
CDKN1A: Cyclin dependent kinase inhibitor 1 A
CDKN1B: Cyclin dependent kinase inhibitor 1 B
CDKN2A: Cyclin dependent kinase inhibitor 2 A
CDKN2B: Cyclin dependent kinase inhibitor 2 B
CDK: Cyclin dependent kinase
HCC: Hepatocellular carcinoma
siTERT: small interference TERT
AURKA: Aurora kinase A
AURKB: Aurora kinase B
DMEM: Dulbecco's Modified Eagle Medium
PI: propidium iodide
SEM: standard error of the mean
EtOH: Ethylene alcohol
TUNEL: Terminal deoxynucleotidyl transferase dUTP nick end labeling
IHC: immunohistochemistry
IPA: Ingenuity pathway analyses
NGS: next generation sequencing
