# Supplementary material for: HKR3 regulates cell cycle through the inhibition of hTERT in hepatocellular carcinoma cell lines
Source: J Cancer. 2020 Feb 10;11(9):2442–52. doi: 10.7150/jca.39380 (PMC7066026; doi:10.7150/jca.39380)
Supplement: Supplementary file 1 — Supplementary figures and table. [file jcav11p2442s1.pdf]

Supplementary figure legends

Supplementary Figure 1. Transcriptomic and Proteomic analysis of siTERT and HKR3 overexpression

(A) In RNAseq analysis, gene changes significantly different pattern from siTERT and HKR3 overexpression groups can be compared with control. (B) In LC/MS analysis, protein changes were identified in siTERT and HKR3 overexpression, significantly. (C) Venn diagram of siTERT and HKR3 overexpression shows the change of various biological processes.

Supplementary Figure 2. Effects of apoptosis by siTERT and HKR3

Cell death was increased in siTERT and HKR3 compared to control.

Supplementary Figure 1

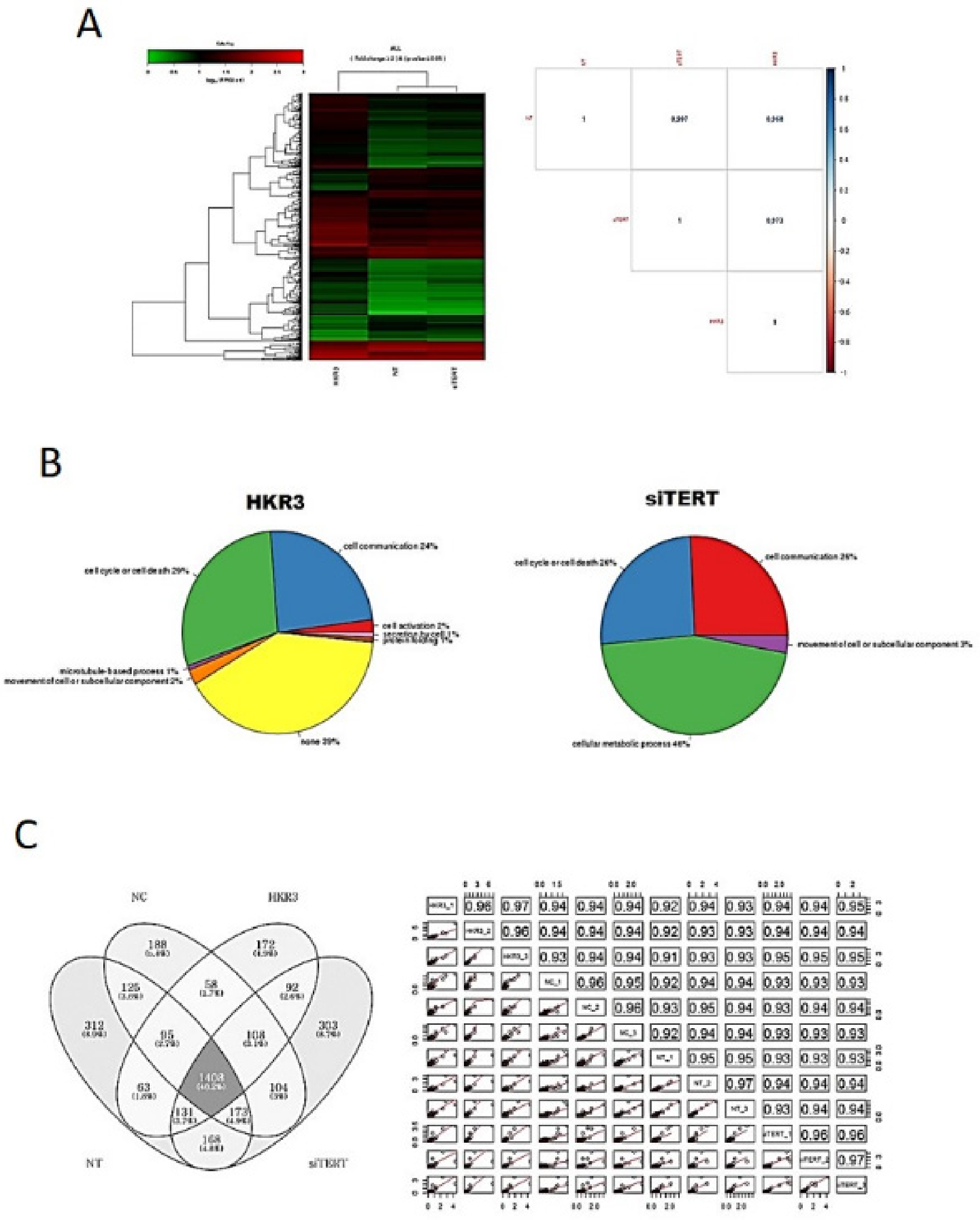

Supplementary Figure 2

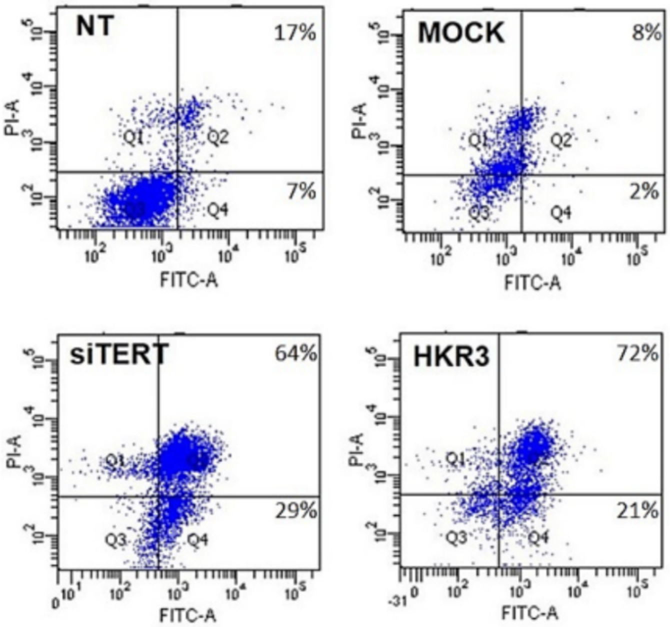

Supplementary Table 1

	Forward	Reverse
**Cyclin A1**	GCCATTAGTTTACCTGGACCCAGA	CACTGACATGGAAGACAGGAACCT
**Cyclin A2**	TACCTGGACCCAGAAAACCA	CACTCACTGGCTTTTCATCTTCT
**Cyclin B1**	ACGAAGGTCTGCGCGTGTT	CCGCTGGCCATGAACTACCT
**Cyclin B2**	TTCAACCCACCAAAACAACA	CTCAGGTGTGGGAGAAGGAC
**Cyclin D1**	AGACCTGCGCGCCCTCGGTG	GTAGTAGGACAGGAAGTTGTTC
**Cyclin E1**	GTCCTGGCTGAATGTATACATGC	CCCTATTTTGTTCAGACAACATGGC
**Cyclin E2**	GGGAAACATTTTATCTTGCACA	CTGCAAGCACCATCAGTGAC
**CDK2**	GCTAGCAGACTTTGGACTAGCCAG	AGCTCGGTACCACAGGGTCA
**CDK4**	CTGGTGTTTGAGCATGTAGACC	AAACTGGCGCATCAGATCCTT
**CDKN1A**	CCTCATCCCGTGTTCTCCTTT	GTACCACCCAGCGGACAAGT
**CDKN1B**	CCGGCTAACTCTGAGGACAC	AGAAGAATCGTCGGTTGCAG
**CDKN2A**	CAACGCACCGAATAGTTACGG	AACTTCGTCCTCCAGAGTCGC
**CDKN2B**	TGGCCGGAGGTCATGATG	GGGCAGCATCATGCACCG
**AURKA**	TTTCCATGATGCTACCAGAGTC	GGGCATTTGCCAATTCTG
**AURKB**	TCTCTAAGGATGGCCCAGAA	GCTCATGAGGACAAGTGCAG
**hTERT**	GGAGCAAGTTGCAAAGCATTG	TCCCACGACGTAGTCCATGTT
**TP53**	TGCAGCTGTGGGTTGATTCC	AAACACGCACCTCAAAGCTGTTC
**MDM2**	CCCTTAATGCCATTGAACCT	CATACTGGGCAGGGCTTATT
**P21**	CAGACCAGCATGACAGATTTC	TTAGGGCTTCCTCTTGGAGA
